# Nitric Oxide Regulates GluA2-Lacking AMPAR Contribution to Synaptic Transmission of CA1 Apical but Not Basal Dendrites

**DOI:** 10.3389/fnsyn.2021.656377

**Published:** 2021-06-03

**Authors:** Violetta O. Ivanova, Pavel M. Balaban, Natalia V. Bal

**Affiliations:** Cellular Neurobiology of Learning Lab, Institute of Higher Nervous Activity and Neurophysiology of the Russian Academy of Science, Moscow, Russia

**Keywords:** AMPA, nitric oxide, hippocampus, synaptic transmission, pyramidal neurons

## Abstract

The mechanisms of synaptic plasticity differ in distinct local circuits. In the CA1 region of the hippocampus, the mechanisms of long-term potentiation (LTP) at apical dendrites in *stratum radiatum* and basal dendrites in *stratum oriens* involve different molecular cascades. For instance, participation of nitric oxide in LTP induction was shown to be necessary only for apical dendrites. This phenomenon may play a key role in information processing in CA1, and one of the reasons for this difference may be differing synaptic characteristics in these regions. Here, we compared the synaptic responses to stimulation of apical and basal dendrites of CA1 pyramidal neurons and found a difference in the current–voltage characteristics of these inputs, which is presumably due to a distinct contribution of GluA2-lacking AMPA receptors to synaptic transmission. In addition, we obtained data that indicate the presence of these receptors in pyramidal dendrites in both *stratum radiatum* and *stratum oriens*. We also demonstrated that inhibition of NO synthase reduced the contribution of GluA2-lacking AMPA receptors at apical but not basal dendrites, and inhibition of soluble guanylate cyclase did not affect this phenomenon.

## Introduction

The CA1 region of the hippocampus is a prevailing part of the brain for studying the phenomena of synaptic plasticity. This region with its widespread projections (Cenquizca and Swanson, 2007) is a key structure for the propagation of signals from the hippocampus to other parts of the brain. The apical and basal dendrites of the CA1 pyramidal cells extend in two main directions: *stratum radiatum/stratum lacunosum moleculare* and *stratum oriens*, respectively. Most of the excitatory inputs to CA1 pyramidal neurons originate from CA3 pyramidal cells through their ipsilateral Schaffer collaterals and contralateral commissural fibers to *str. radiatum* (Van Strien et al., 2009), as well as from the entorhinal cortex through the perforant pathway into the *stratum lacunosum moleculare* (Masurkar et al., 2017). Synaptic plasticity at basal dendrites may be an important part of information processing in CA1, since synaptic plasticity of CA1 apical dendrite synapses can be homeostatically regulated by the cell-wide history of synaptic activity (metaplasticity) including the activity of basal dendrites (Hulme et al., 2012). While synaptic plasticity at the apical dendrites of CA1 pyramidal neurons has been extensively studied, relatively little is known whether the mechanisms of synaptic plasticity are the same at basal dendrites.

Synaptic plasticity at the basal and apical dendrites of the hippocampal CA1 region has some similarities (Bradshaw et al., 2003), but the clear spatial separation of these synapses, combined with differences in the innervation of *str. radiatum* and *str. oriens*, suggests that the molecular mechanisms of plasticity may vary significantly. For instance, knockout of both the endothelial and neuronal forms of nitric oxide synthase (NOS) caused a drop in amplitude responses after induction of long-term potentiation (LTP) only at apical dendrites (Son et al., 1996), but not at basal dendrites, which is consistent with other data regarding the involvement of NOS in synaptic plasticity in *str. radiatum* and *str. oriens* (Haley et al., 1996). In addition, differences were found in the molecular cascades dependent on nitric oxide (NO): inhibition of cyclic guanosine monophosphate (cGMP), cGMP-dependent protein kinase (PKG), and soluble guanylyl cyclase (sGC) during LTP induction were demonstrated to be more effective in blocking LTP in *str. radiatum* than in *str. oriens* (Son et al., 1998). Authors suggest that this difference is due to the fact that endothelial NO synthase (eNOS) is not present in *str. oriens*, which is the main source of nitric oxide in hippocampal LTP according to O’Dell et al. (1994). However, these data contradict other studies. For instance, it was shown that there is more eNOS in *str. radiatum* than in *str. oriens* due to the higher density of blood vessels (Blackshaw et al., 2003) and that LTP maintenance requires involvement of at least one form of NOS (Son et al., 1996). It is important to note that LTP sensitivity to NO in *stratum radiatum* also depends on the stimulation protocol for LTP induction (Lu et al., 1999; Bal et al., 2017; Maltsev et al., 2019). Taken together, these data indicate that the mechanism of LTP at basal dendrites of CA1 pyramidal cells is likely to be NO-independent. However, it should be noted that the neural NOS is still present in *str. oriens* (O’Dell et al., 1994; Blackshaw et al., 2003), and application of NO donors caused cGMP production in *str. oriens* (Bartus et al., 2013), which suggests that nitric oxide has some other functions in this region.

One of the key participants in LTP is the α-amino-3-hydroxy-5-methyl-4-isoxazolepropionic acid receptor (AMPAR) which is the main provider of excitatory transmission in the mammalian CNS (Malinow and Malenka, 2002; Diering and Huganir, 2018). Most AMPARs are heterotetramers combined from the GluA1, GluA2, GluA3, and GluA4 subunits. In the adult brain, almost all GluA2 subunit mRNA undergoes posttranscriptional editing, which leads to a replacement of the neutral amino acid glutamine with positively charged arginine in the polypeptide chain of the subunit. This replacement alters the electrophysiological properties of GluA2-containing AMPARs and makes them impermeable to calcium (Higuchi et al., 1993). Nitric oxide was shown to affect the incorporation of different AMPAR subunits to the cell membrane *via* several different pathways (for review, see Ivanova et al., 2020). Thus, the interaction of nitric oxide with AMPAR subunits could be crucial in the trafficking of GluA2-lacking AMPARs [calcium-permeable (CP-AMPARs)].

In our study, we demonstrate the differences in AMPAR-NO interactions between apical and basal dendrites. We show that the contribution of CP-AMPARs to synaptic transmission in apical dendrites is higher than in basal dendrites, and the NO synthase blockade flattens this difference. This effect does not involve sGC-dependent cascades. Our results confirm previous studies demonstrating different NO-dependent mechanisms in the apical and basal dendrites of CA1 pyramidal neurons.

## Materials and Methods

### Animals and Ethical Approval

All experiments followed the European Convention for the Protection of Vertebrate Animals used for Experimental and other Scientific Purposes 1986 86/609/EEC and were approved by the Ethical Committee of the Institute of Higher Nervous Activity and Neurophysiology, Russian Academy of Sciences (IHNA RAS). The mice were purchased from the Nursery for laboratory animals of the Branch of the Institute of Bioorganic Chemistry of the Russian Academy of Sciences in Pushchino. The mice were maintained in a temperature-controlled vivarium (22 ± 2°C) under a 12-h light/dark cycle (lights on at 08.00 h) with food and water *ad libitum*. All efforts were made to minimize animal suffering and to reduce the number of animals used.

### Slice Preparation

Horizontal brain slices (300 μm thick) containing the ventral hippocampus and entorhinal cortex were prepared from the brains of 25–35-day-old C57Bl/6 female and male mice killed by decapitation. The slicing chamber contained an oxygenated ice-cold solution (modified from Dugue et al., 2005) composed of the following (in mM) K-gluconate, 140; N-(2-hydroxyethyl) piperazine-N′-ethanesulfonic acid (HEPES), 10; Na-gluconate, 15; ethylene glycol-bis(2-aminoethyl)-N,N,N′,N′-tetraacetic acid (EGTA), 0.2; and NaCl, 4 (pH 7.2 with KOH). Brain slices were cut using a Vibratome (Leica VT1000S, Germany). Slices were incubated for at least 40 min at 35°C before being stored at room temperature in artificial CSF (ACSF) containing the following (in mM): NaCl, 125; NaHCO_3_, 25; KCl, 2.5; NaH_2_PO_4_, 1.25; MgCl_2_, 3.9; CaCl_2_, 1; and glucose, 25; bubbled with 95% O_2_, and 5% CO_2_.

### Electrophysiology

Electrophysiological recording was performed in an acrylic glass perfusion chamber (Luigs and Neumann, Germany) with the bath temperature kept at 30 ± 2°C and perfused at a constant rate of 3 ml/min. Patch electrodes (resistance 4–5 MΩ) were pulled from borosilicate capillary glass (Narishige PC-100 Puller, Japan) and were filled with either a polyamine-free or a polyamine-containing solution. The polyamine-free solution consisted of the following (in mM): Cs-gluconate, 110; CsCl, 30; HEPES, 10; NaCl, 8; EGTA, 0.2; MgATP, 4; Na_3_GTP, 0.3; and phosphocreatine, 10 (pH 7.3 with CsOH) osmolarity ~290 mOsm. The polyamine-containing solution was identical except for the addition of 10 μM spermine.

CA1 pyramidal cells were identified visually using an Olympus microscope fitted with infrared differential interference contrast optics (Olympus BX51WI). Whole-cell recordings from these neurons were made in a voltage-clamp mode using the ELC-03XS amplifier (NPI Electronic, Tamm, Germany) and Clampex software (Axoclamp, Molecular Devices). Cells were held at −70 mV. Cells with unhealthy morphology and resting membrane potential above −50 mV (before correction for the liquid junction potential) were excluded from the experiments. To evoke synaptic current, glass electrodes filled with ACSF were placed in the dendritic region of *stratum radiatum* and *stratum oriens*, ~50–100 μm from the cell body, to stimulate the inputs at interstimulus intervals of 6 s. Inhibitory synaptic transmission was blocked during recordings by adding 50 μM picrotoxin to the perfusion ACSF. In all the experiments except AMPA/NMDA ratio measurements, the NMDA-mediated component was blocked by adding 50 μM APV to the ACSF. For the experiments with NOS inhibition, slices were incubated for 40–120 min in L-NAME, 15 min in 3-bromo-7-nitroindazole or carboxy-PTIO before being placed in the perfusion chamber. Whole-cell recordings typically started 5–10 min after break-in, when the balance between intracellular milieu and patch solution was established and a steady-state current was reached, except the experiments with GluR2-lacking AMPA receptor inhibition. The stimulation intensity was adjusted to produce an EPSC with an amplitude of ~50 pA at the beginning of each recording. The experiments were not started if there was an unstable baseline. Series resistance was monitored, and data from cells in which series resistance varied by >15% during recording were discarded from the analysis. In all the experiments, the command voltage was corrected for the liquid junction potential (−10 mV).

#### GluR2-Lacking AMPA Receptor Inhibition

Experiments were performed using QX-314-containing spermine-free intracellular solution. Recording started after the holding current stabilizes (1–2 min after the beginning of whole-cell recording). The amplitude of test responses stabilizes after 15–20 min of recording. The GluR2-lacking AMPA receptor antagonist Naspm (200 μM) was applied 30 min after the start of the recording. For analysis, 10 successive responses were averaged and normalized to the mean EPSC amplitude obtained between 20 and 30 min of the recording session. The degree of the blockade was evaluated as the ratio of the average steady-state current amplitude without and after Naspm application.

#### Current–Voltage Relationship

The experiments started after baseline stabilization (~100 sweeps). For each holding potential point, at least 20 sweeps were collected. Responses were averaged and normalized to the mean EPSC amplitude obtained at −70 mV. To evaluate differences, the normalized values at +50 mV were compared.

#### Rectification Index (RI)

Experiments started after baseline stabilization (~100 sweeps). For the −70-mV and +35-mV holding points, at least 30 and 50 sweeps were collected, respectively. The RI was calculated as the ratio of EPSCs measured at −70 mV and +35 mV (EPSC_-70_/EPSC_+35_). For each +35-mV point, only the last 30 sweeps were analyzed due to potential space clamp problems.

To test synapses for *polyamine-dependent facilitation (PdF)*, we applied four stimuli with an interstimulus interval of 33 ms ×40 for each of the inputs, 5 and 20 min after whole-cell patch formation. To evaluate changes in PdF, we normalized each of the EPSCs to the first in the train, averaged the obtained values, and separately compared the data for apical and basal dendrites in different conditions. For PdF analysis, an additional selection criterion was applied: traces with undetectable peaks of the first EPSC in the train were retracted from the analysis. The number of the retracted traces was <1% of the total number of traces.

*Paired-pulse ratio* was monitored by applying two stimuli with a 50-ms interstimulus interval. The experiments started after baseline stabilization (~100 sweeps), and at least 20 sweeps were collected.

#### AMPA/NMDA Current Ratio

The AMPA/NMDA ratio was measured in Mg^2+^-free ACSF. AMPA and NMDA receptor-mediated EPSCs were pharmacologically isolated by sequential bath application of APV and CNQX, respectively. First, the compound AMPAR and NMDA-mediated current was recorded in Mg^2+^-free ASCF. After collecting at least 100 sweeps, the AMPA-mediated component was blocked by application of 50 μM CNQX. An additional 100 sweeps of the NMDA-mediated currents were collected and the NMDA nature of these currents was confirmed by subsequent application of APV. The AMPA-mediated component was obtained by subtraction of the averaged NMDA-mediated currents from the averaged compound response. For subsequent analysis, the mean amplitude of the AMPA currents was normalized to the amplitude of the NMDAR EPSCs.

Cells that did not correspond to the standard criteria of electrophysiological properties, such as input resistance, series resistance, and baseline holding current, were excluded from the analysis.

### Drugs

ODQ from Sigma-Aldrich was prepared as a 25-mM stock solution in DMSO and diluted down to achieve a final bath concentration of 30 μM. 3-Bromo-7-nitroindazole (3-Br-7-ni; Enzo Life Sciences) was dissolved as a 100-mM stock in DMSO and diluted down to achieve a final bath concentration of 50 μM. Nω-Nitro-L-arginine methyl ester hydrochloride (L-NAME; Sigma-Aldrich) was prepared as a 200-mM stock in milli-Q water diluted down to achieve a final bath concentration of 200 μM. Carboxy-PTIO (2-(4-Carboxyphenyl)-4,4,5,5-tetramethylimidazoline-1-oxyl-3-oxide potassium salt) from Enzo Life Sciences was prepared as a 100-mM stock in DMSO and diluted down to achieve a final bath concentration of 50 μM. Naspm (1-naphthylacetyl spermine) was prepared as a 100-mM stock in milli-Q water and diluted down to achieve a final bath concentration of 200 μM (Tocris Bioscience). Picrotoxin from Sigma-Aldrich was prepared as a 100-mM stock in DMSO and diluted down to achieve a final bath concentration of 50 μM. DL-2-Amino-5-phosphonopentanoic acid sodium salt (APV) from Tocris was prepared as a 100-mM stock in milli-Q water and diluted down to achieve a final bath concentration of 50 μM. CNQX disodium salt from Tocris Bioscience was prepared as a 100-mM stock in milli-Q water and diluted down to achieve a final bath concentration of 50 μM.

### Statistical Analysis

**Figure 1:**
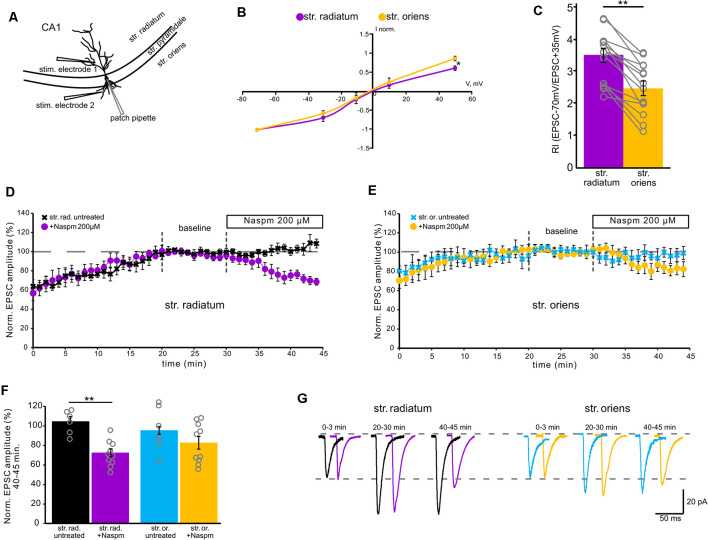
Contribution of calcium-permeable AMPA receptors to synaptic transmission. **(A)** A schematic representation of the location of the stimulating electrode. **(B)** Current–voltage characteristics of apical (purple) and basal (orange) inputs. *n* = 7 cells from four animals, *p ≤ 0.05. **(C)** Comparison of rectification indices in *stratum radiatum* (purple) and *stratum oriens* (orange). Open gray circles represent individual data points, *n* = 14 cells from nine animals, **p ≤ 0.005. **(D)** The time course of averaged EPSC amplitude changes at apical inputs during washout of polyamine (black, *n* = 6 cells from three animals) and subsequent Naspm (200 μM) application (purple, *n* = 10 cells from eight animals), when CA1 pyramidal neurons were dialyzed with a polyamine-free intracellular solution. For analysis, 10 successive responses were averaged and normalized to the mean EPSC amplitude obtained between the 20th and 30th minutes of the recording session (baseline marked with gray dashed lines). **(E)** The same time course as **(D)** but for basal inputs. Blue, untreated cells, *n* = 6 cells from five animals. Orange, cells treated with Naspm (200 μM), (*n* = 10 cells from eight animals). **(F)** A histogram demonstrating normalized EPSCs for the last 5 min of the curves D and E. Open gray circles represent individual data points, **p ≤ 0.005. **(G)** Representative averaged traces at the indicated times of the curves D and E are shown in black and purple for apical dendrites, and in blue and orange for basal dendrites.

**Figure 2:**
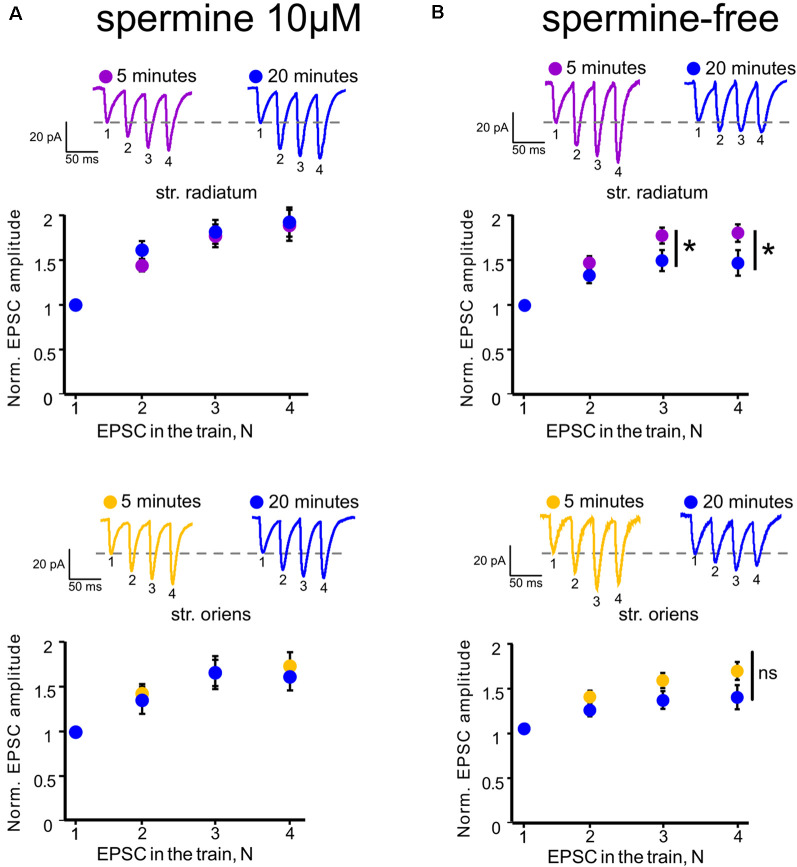
Polyamine-dependent facilitation at the synapses of CA1 pyramidal cells. **(A)** Polyamine-dependent facilitation at apical (upper) and basal (lower) dendrites recorded with spermine-containing intracellular solution 5 and 20 min after whole-cell patch establishment, *n* = 6 cells from four animals. **(B)** Polyamine-dependent facilitation at apical (upper) and basal (lower) dendrites recorded with spermine-free intracellular solution 5 and 20 min after whole-cell patch establishment, *n* = 8 cells from five animals, *p ≤ 0.05; ns, nonsignificant. Averaged traces of responses to stimuli with a 33-ms interstimulus interval at the indicated times are displayed above each graph. EPSC numbering corresponds to the x-axis of each graph.

**Figure 3:**
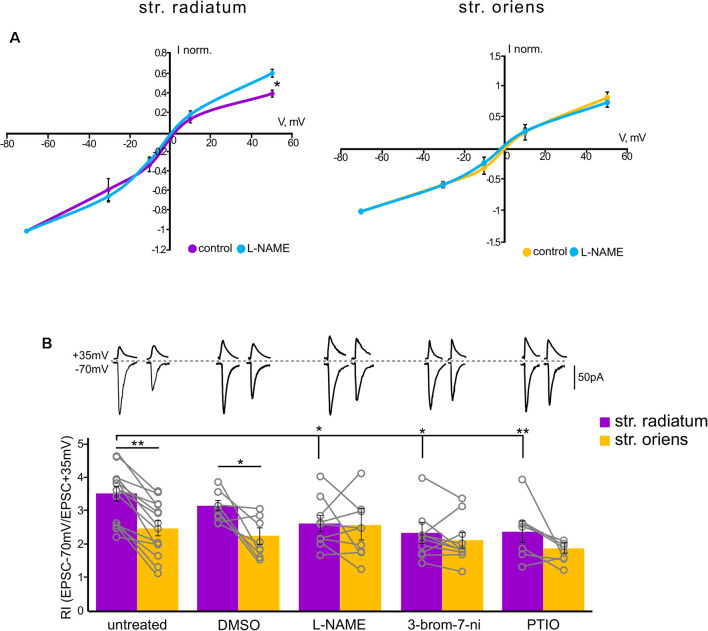
Rectification properties of the apical and basal inputs under the NO-synthase inhibitors. **(A)** Current–voltage characteristics of apical (left) and basal (right) inputs in control cells (*n* = 7) and under nitric oxide synthase (NOS) blockade (*n* = 7), four animals, *p ≤ 0.05. **(B)** Comparison of rectification indices in *stratum radiatum* (purple) and *stratum oriens* (orange) in control cells (*n* = 14 cells from nine animals) and under treatment with DMSO (*n* = 8 cells from four animals), L-NAME (*n* = 8 cells from six animals), 3-bromo-7-nitroindazole (*n* = 10 cells from seven animals), and PTIO (*n* = 7 cells from four animals). Top, example traces at +35 mV and −70 mV. *p ≤ 0.05, **p ≤ 0.005. Open gray circles represent individual data points.

**Figure 4:**
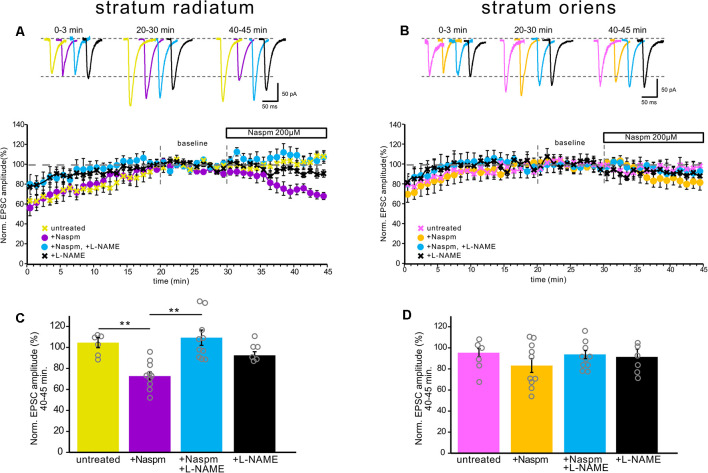
NOS inhibition prevented CP-AMPAR current blockade by Naspm in the apical dendrites, but not in the basal dendrites. **(A)** The time course of EPSC amplitude changes in apical inputs during washout of polyamine (yellow, *n* = 6 cells from five animals), Naspm (200 μM) application (purple, *n* = 10, eight animals), and under NOS inhibition (black, *n* = 6 cells from four animals) and under NOS inhibition with Naspm application (blue, *n* = 10 cells from eight animals). For analysis, 10 successive responses were averaged and normalized to the mean EPSC amplitude obtained between 20 and 30 min of the recording session (baseline marked with gray dashed lines). Top, example traces. **(B)** The same time course as **(A)** but for basal inputs. Pink, untreated cells, *n* = 6 cells from five animals. Yellow, cells treated with Naspm, *n* = 10 cells from eight animals. Blue, cells treated with L-NAME and Naspm, *n* = 10 cells from eight animals. Black, cells treated with L-NAME, *n* = 6 cells from four animals. Top, example traces. **(C,D)** Histograms demonstrating normalized EPSCs for the last 5 min of the curves A and B, respectively. Open gray circles represent individual data points, **p ≤ 0.005.

**Figure 5:**
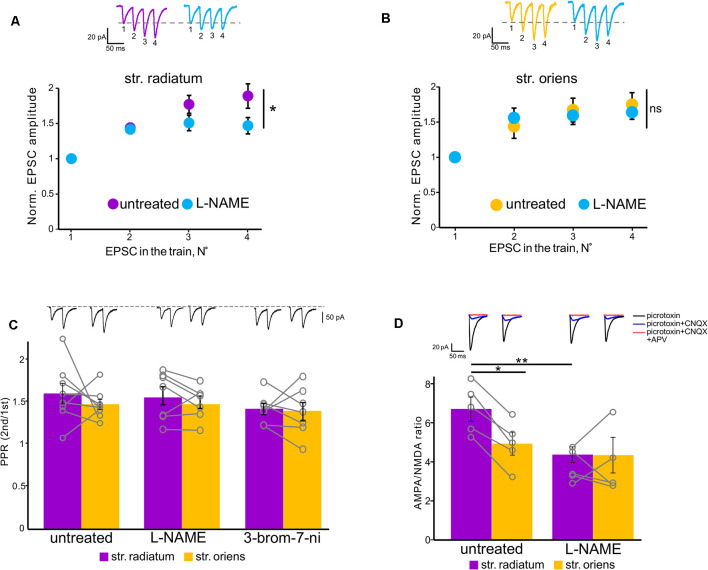
NOS inhibition affected polyamine-dependent facilitation in the apical but not basal dendrites. **(A)** Facilitation at apical dendrites recorded with spermine-containing intracellular solution in untreated cells (purple, *n* = 6 cells from four animals) and under NOS blockade (orange, *n* = 6 cells from four animals). *p ≤ 0.05. Top, example traces. **(B)** Basal dendrite responses recorded with spermine-containing intracellular solution in untreated cells (purple, *n* = 7 cells from four animals) and under NOS blockade (orange, *n* = 7 cells from four animals). Top, example traces. EPSC numbering corresponds to the x-axis of each graph. ns, nonsignificant. **(C)** Comparison of paired-pulse ratios in *stratum radiatum* (purple) and *stratum oriens* (orange) in untreated cells and under NOS blockade (control: *n* = 8 cells from five animals; L-NAME: *n* = 7 cells from three animals; 3-bromo-7-ni: *n* = 7 cells from three animals). Open gray circles represent individual data points. Top, example traces. **(D)** Comparison of AMPA-NMDA ratios in *stratum radiatum* (purple) and *stratum oriens* (orange) in untreated cells (*n* = 5 cells from three animals) and under NOS blockade (*n* = 5 cells from three animals). Top, example traces. *p ≤ 0.05, **p ≤ 0.005.

**Figure 6:**
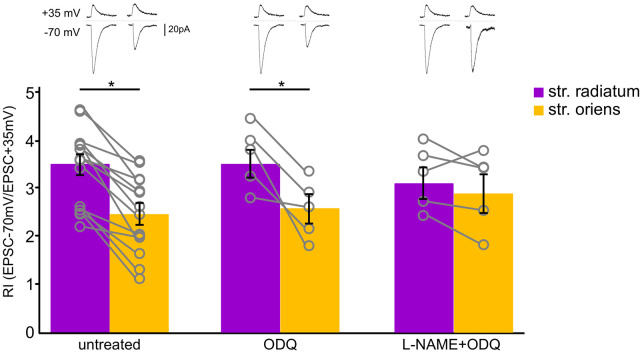
Soluble guanylyl cyclase inhibition did not affect CP-AMPAR contribution to synaptic transmission of CA1 pyramidal cells. Comparison of rectification indices in *stratum radiatum* (purple) and *stratum oriens* (orange) in untreated cells (*n* = 14 cells from nine animals) and under treatment with ODQ (30 μM, *n* = 5 cells from three animals) and L-NAME (200 μM, *n* = 5 cells from three animals). Top, example traces. *p ≤ 0.05.

Results are presented as mean ± standard error (S.E.M.) of *n* cells. All statistical tests were performed using SigmaPlot 11.0 (Systat Software Inc., USA). For pairwise comparisons (Figures 1B,C), one-way ANOVA was used. For multiple comparisons (Figures 1F, 3A,B, 4C,D, 5C,D, 6, Supplementary Figure 1), we used two-way ANOVA. A significant main effect or interaction was followed by *post-hoc* comparison using Multiple Comparisons vs. Control Group (Holm–Sidak test); for between-subject analysis, the untreated cells were scored as “Control Group.” For Figures 2, 5A,B, and Supplementary Figure 2, we used two-way repeated-measures ANOVA. A significant main effect or interaction was followed by *post-hoc* comparisons using Multiple Comparisons vs. Control Group (Holm–Sidak Test). For between-subject analysis, the control cells were scored as “Control Group,” and for within-subject analysis all measurements were compared to the first EPSC in the train. A probability level of 0.05 or less was considered statistically significant (*p ≤ 0.05, **p ≤ 0.005).

## Results

### Calcium-Permeable AMPA Receptors Contribute More to Synaptic Transmission at Apical Dendrites of CA1 Pyramidal Neurons Than at Basal Dendrites

First, to evaluate the contribution of GluA2-lacking AMPARs to currents in apical and basal compartments of CA1 pyramidal neurons, we measured the RI by stimulating apical and basal dendrites with glass electrodes located as shown in Figure 1A. The rectification index reflects inward rectification of GluA2-lacking AMPARs and is scored as the ratio of the current amplitude measured at −70 mV and +35 mV (EPSC_–70 mV_/EPSC_+35 mV_). We found that the rectification index in responses to stimulation of apical dendrites (RI = 3.4 ± 0.2) is significantly higher than in responses to stimulation of basal dendrites (RI = 2.5 ± 0.2; *p* = 0.005, one-way ANOVA, *n* = 14, Figure 1C). The current–voltage (IV) characteristics for each of the inputs supported the difference revealed by RI measurement; the apical IV demonstrated significantly higher inward rectification (*p* = 0.035, one-way ANOVA, *n* = 7, Figure 1B). The most common approach to detecting the contribution of GluA2-lacking AMPARs to synaptic transmission is to test sensitivity to CP-AMPAR antagonists, for example, 1-naphthylacetyl spermine trihydrochloride (Naspm). Therefore, we tested whether the responses to stimulation of apical and basal inputs are sensitive to extracellular Naspm (200 μM) application (Figures 1D–G). To evaluate the degree of amplitude reduction, we averaged the values from each cell in the 5-min segment at the end of the recordings (Figure 1F). We observed a decrease in AMPAR currents in both of the inputs (73% ± 4 of the baseline in *str. radiatum* and 83% ± 6.5 in *str. oriens*, *n* = 10), which may indicate the presence of CP-AMPARs in the basal and apical dendrites of CA1 pyramidal neurons. However, comparison to the untreated cells (*n* = 6) showed significant difference only in apical (73% ± 4 vs. 109% ± 7, *p* = 0.001, two-way ANOVA) but not in basal inputs (83% ± 6.5 vs. 94% ± 4, *p* = 0.354, two-way ANOVA). Even at physiological resting membrane potentials (~−70 mV), a substantial portion of GluA2-lacking channels could still be blocked by polyamines during single unitary EPSCs. Thus, we used a spermine-free intracellular solution in these experiments. As a result of the endogenous polyamine washout from the dendrites, the amplitude of responses increased up to 20 min. To avoid subsequent spike generation, we added 30 μM QX-314 to the intracellular solution. Due to the different morphology of apical and basal dendrites (Benavides-Piccione et al., 2020), the response amplitude at the basal inputs tends to stabilize earlier than at the apical inputs.

In addition, Rozov and colleagues have shown previously that application of several high-frequency stimuli to the input with CP-AMPARs causes relief of the polyamine block which, in turn, results in polyamine-dependent facilitation (PdF; Rozov and Burnashev, 1999; Rozov et al., 2018). We applied a similar protocol to the apical and basal inputs of CA1 pyramidal cells and found that this type of short-term plasticity was characteristic for synapses of these cells. We applied four stimuli with an interstimulus interval of 33 ms to each of the inputs at the beginning of the recording and 20 min later. We used spermine-free and spermine-containing intracellular solutions in order to test whether polyamine washout would affect the EPSCs. For experiments with polyamine washout, we adjusted the stimuli strength after 20 min to avoid spikes due to potential space clamp problems. Figures 2A,B demonstrate the results of these experiments. When we used a spermine-free intracellular solution, the 3rd and 4th EPSCs in the train were significantly lower 20 min after polyamine washout than at the beginning of the recording at apical dendrites (*p* = 0.037 for the 3rd EPSCs and *p* = 0.014 for the 4th EPSCs, two-way RM ANOVA, *n* = 8), and we observed the same tendency at basal dendrites (*p* = 0.117 for the 3^d^ EPSCs and *p* = 0.066 for the 4th EPSCs, two-way RM ANOVA, *n* = 8), whereas with the presence of polyamines in the patch pipette we did not observe such differences (apical: *p* = 0.46 for the 3rd EPSCs and *p* = 0.101 for the 4th EPSCs; basal: *p* = 0.47 for the 3rd EPSCs and *p* = 0.26 for the 4th EPSCs, two-way RM ANOVA, *n* = 6). Thus, we showed polyamine-dependent facilitation at the synapses of CA1 pyramidal cells, which indicates the presence of CP-AMPARs at both apical and basal dendrites of these cells. However, experiments measuring the current–voltage characteristics of these inputs revealed that the CP-AMPA receptors contribute more to synaptic transmission at apical dendrites of CA1 pyramidal neurons than basal dendrites.

### Nitric Oxide Synthase Blockade Alters CP-AMPAR Contribution to Currents in Apical but Not Basal Dendrites

Since nitric oxide can affect the incorporation of CP-AMPARs into the postsynaptic membrane of cells, we tested whether the blockade of its synthesis by various NO synthase inhibitors affects the characteristics of the apical and basal synapses of CA1 pyramidal cells. Figure 3B shows the rectification indices of the apical and basal inputs under the NO-synthase inhibitor L-NAME (200 μM), the selective inhibitor of neuronal NO-synthase 3-bromo-7-nitroindazole (50 μM), and the NO scavenger carboxy-PTIO (50 μM). The latter two inhibitors were dissolved in DMSO, and we also tested the effect on RI of DMSO alone (25 μl). A decrease in the intracellular concentration of nitric oxide significantly reduced RIs at the apical dendrites compared to control cells (DMSO: 3.1 ± 0.1, *p* = 0.321, *n* = 8; L-NAME: 2.6 ± 0.2, *p* = 0.025, *n* = 8; 3-br-7-ni: 2.4 ± 0.3, *p* = 0.007, *n* = 10; PTIO: 2.4 ± 0.3, *p* = 0.002, *n* = 7, two-way ANOVA), but not basal dendrites (DMSO: 2.1 ± 0.2, *p* = 0.28, *n* = 8; L-NAME: 2.6 ± 0.4, *p* = 0.78, *n* = 8; 3-br-7-ni: 2.1 ± 0.2, *p* = 0.24, *n* = 10; PTIO: 1.8 ± 0.1, *p* = 0.07, *n* = 7, two-way ANOVA), in various ways, thus leveling the significant difference between these two inputs. The current–voltage characteristics of the studied inputs under NOS blockade by L-NAME demonstrated a loss of inward rectification at synaptic inputs in apical but not basal dendrites (*p* = 0.031 for apical, *p* = 0.4 for basal, two-way ANOVA, *n* = 7, Figure 3A). We also compared the AMPA/NMDA ratio at apical and basal dendrites with or without NOS inhibition (Figure 5D). In control cells, this ratio was significantly higher at apical dendrites than at basal dendrites (6.7 ± 0.6 vs. 4.9 ± 0.6, *n* = 5, *p* = 0.049, two-way ANOVA), and this difference disappeared after NOS inhibition (4.3 ± 0.8 vs. 3.9 ± 0.8, *n* = 5, *p* = 0.788, two-way ANOVA), supporting the results of the experiments with RI measurement.

Additionally, NOS inhibition prevented the drop in the current amplitude during CP-AMPAR blockade by Naspm in *str. radiatum* (109% ± 7.3 vs. 3% ± 4, *n* = 10, *p* = 0.001, two-way ANOVA, Figure 4C); however, in *str. oriens* we did not find a significant difference in the last 5 min of recording (89.4% ± 3.2 vs. 83% ± 6.5, *n* = 10, *p* = 0.264, two-way ANOVA, Figure 4D). Interestingly, the increase in the current amplitude at the beginning of the recording, which is associated with polyamine washout in both *str. radiatum* and *str. Oriens*, persisted after incubation in L-NAME (Figures 4A,B). This might be due to either specific mechanisms associated with polyamine washout or the possibility of NO-dependent regulation of CP-AMPAR sensitivity to polyamines. In the latter case, polyamines could still block the receptor pore but the blockade is more easily relieved which causes smaller EPSC increase and faster baseline stabilization.

Next, we tested how the decrease of nitric oxide in the cell would affect polyamine-dependent facilitation at the studied inputs in the presence of spermine in the recording pipette (Figures 5A,B). NOS inhibition significantly affects the 4th EPSCs at apical dendrites (*p* = 0.006, two-way RM ANOVA, *n* = 7), but not at basal dendrites (*p* = 0.499, two-way RM ANOVA, *n* = 7). PdF recording was performed at the fifth minute of recording after a stable baseline was reached. Considering the wide range of nitric oxide action in the presynapse of cells (Hardingham et al., 2013), we tested whether the discovered effect was due to the presynaptic effect of nitric oxide. One of the possible approaches for evaluation of the presynaptic contribution to synaptic transmission is paired-pulse ratio measurement (Schulz et al., 1994; Christie and Jahr, 2006). Recording of paired-pulse ratio at the synapses of CA1 pyramidal cells during NOS inhibition did not reveal significant differences in these cells compared to control cells (control: 1.6 ± 0.1 in *str. radiatum*, and 1.4 ± 0.06 in *str. oriens*, *n* = 8; L-NAME: 1.5 ± 0.1 in *str. radiatum*, and 1.4 ± 0.07 in *str. oriens*, *n* = 7; 3-bromo-7-ni: 1.4 ± 0.06 in *str. radiatum*, and 1.4 ± 0.1 in *str. oriens*, *n* = 7). Thus, the results suggest that the disappearance of PdF in *str. radiatum* after inhibition of nitric oxide synthesis was associated with the interaction of nitric oxide with CP-AMPARs.

We examined whether the sGC-dependent pathway is involved in this interaction by treating slices with the sGC inhibitor ODQ (30 μM). We measured RIs under this condition and found that sGC inhibition did not affect the RIs of neither apical (RI = 3.5 ± 0.2, *n* = 5) nor basal (RI = 2.5 ± 0.3, *n* = 5) inputs (Figure 6, *p* = 0.027, two-way ANOVA). Next, we blocked NOS and sGC simultaneously and found that treating with L-NAME also leveled the RIs of apical (RI = 3.3 ± 0.3, *n* = 5) and basal (RI = 3 ± 0.4) inputs, as in the cells treated with the NOS inhibitors alone (*p* = 0.680, two-way ANOVA).

## Discussion

In the present study, we described data supporting the presence of CP-AMPARs not only in the apical dendrites of CA1 pyramidal cells but also in the basal dendrites. Recording of basic transmission during whole-cell patch-clamp with spermine-free intracellular solution showed a gradual increase in the EPSC amplitudes at both inputs (Figures 1D,E), which is associated with the release of GluR2-lacking AMPARs from the polyamine block and leading to an increase in the conductance of these receptors (Rozov et al., 2012). This growth does not occur with 10 μM spermine in the patch pipette (Supplementary Figure 3). GluR2-lacking AMPAR blockade decreased the response amplitudes significantly in the apical dendrites and insignificantly in the basal dendrites. In addition, application of high-frequency stimulation to the inputs revealed a significant polyamine-dependent facilitation (Rozov and Burnashev, 1999; Rozov et al., 2018) in *str. radiatum*, while in *str. oriens* we observed only a tendency (Figure 2). However, taking into account that basal dendrites are located closer to the soma than apical dendrites, polyamines are washed out *via* the patch pipette faster. According to this, PdF at the basal inputs should slightly decrease by the fifth minute after whole-cell patch formation. Indeed, if the recording starts at ~1 min, the p-value for the 4th EPSC decreases in basal dendrites (Supplementary Figure 2, *p* = 0.024). However, we observed a significantly higher contribution of CP-AMPARs to glutamatergic synaptic transmission in *stratum radiatum*, than in *stratum oriens* by measuring the rectification index (Figure 1C), which suggests that physiology or number of the receptors differs in these compartments.

The presence of GluA2-lacking AMPARs in CA1 cells prompts the question of their localization, as studies have shown different data on their presence in the postsynapse after LTP induction (Plant et al., 2006; Adesnik and Nicoll, 2007; Moult et al., 2010). Some studies have demonstrated that GluA2-lacking AMPARs constitute a small subpopulation of the synaptic AMPA receptors in non-potentiated CA1 pyramidal neurons in adult rodents (Rozov et al., 2012; Mattison et al., 2014), and in other studies, it was shown that GluR2-lacking AMPARs in pyramidal cells in the hippocampus are replaced with GluR2-containing AMPARs in mature animals (Ho et al., 2007; Malkin et al., 2016). This discrepancy can be explained by the presence of polyamines in the patch pipette which affects the ability of GluA2-lacking AMPAR antagonists to block them (Rozov et al., 2012). In addition, the presence of polyamines in the pipette also determines the rectification characteristics of synapses with GluR2-lacking AMPARs in their membrane (Kamboj et al., 1995): the current–voltage characteristics of such synapses in the absence of spermine are linear, as in mutant GluA1−/− mice (Rozov et al., 2012).

The polyamine concentration in the patch pipette varies in different studies (Rozov et al., 2012; Mattison et al., 2014; Malkin et al., 2016). The precise concentration of intracellular free polyamines in CA1 pyramidal cells is unknown; however, it is known that the concentration varies in different regions of the rat brain (Shaskan et al., 1973), as well as in other mammals (Igarashi and Kashiwagi, 2010). In our study, we used 10 μM spermine in the intracellular solution to record the rectification properties of inputs. One can argue that the spermine concentration in our experiments was insufficient to successfully block GluR2-lacking AMPARs; however, the experiments with polyamine-dependent facilitation (Figure 2) indicated the opposite: 10 μM spermine in the patch pipette prevented polyamine washout from the dendritic compartments. Moreover, using 10 μM spermine-containing intracellular solution does not cause an increase in EPSC amplitude (Supplementary Figure 3). In addition, Hu et al. (1994) showed that 100 μM spermine inhibits [3H]L-citrulline formation, which reflects NOS activity, by ~60% in cerebellar cells, whereas 10 μM only slightly inhibits this reaction, Figure 1). This might indicate that high spermine concentration in the patch pipette can cause NOS inhibition. Indeed, when 100 μM spermine was added to the patch pipette, the difference in RIs between apical and basal dendrites of CA1 pyramidal cells was not statistically significant (apical: 3 ± 0.4; basal: 2.3 ± 0.2, *n* = 7, *p* = 0.12, two-way ANOVA; Supplementary Figure 1), as in the case of NOS blockade.

Nitric oxide was shown to be involved in LTP maintenance in *str. radiatum*, but not in *str. Oriens*; however, the presence of nNOS was shown in both *str. radiatum* and *str. oriens* (see the “Introduction” section). We assumed that this contrast was due to the difference in the modulation of synaptic characteristics by nitric oxide in these areas. In particular, nitric oxide could differently affect the CP-AMPAR contribution to synaptic transmission of apical and basal dendrites. We found that NOS inhibition by two different inhibitors and treatment with an NO scavenger reduced inward rectification and caused a drop in the rectification index at apical dendrites (Figure 3), which reflected a decrease in the contribution of GluR2-lacking AMPARs to the currents in these synapses. In addition, NOS inhibition prevented the decrease in response amplitude under Naspm treatment (Figure 4) and reduced the polyamine-dependent facilitation (Figures 5A,B), whereas, at basal inputs the NOS inhibition did not affect any of the synaptic characteristics. Thus, our data indicate that nitric oxide does not affect the contribution of GluR2-lacking AMPARs to synapses of CA1 pyramidal cell basal dendrites, while inhibition of nitric oxide synthesis significantly reduced the contribution of these receptors to apical dendrite synaptic currents. So far, it is unclear which nitric oxide-dependent mechanism exerts this effect in *str. radiatum*. For instance, the concentration of free nitric oxide in the cell can affect synthesis of intracellular polyamines (Buga et al., 1998; Boucher et al., 1999), which in turn determines the conductivity of GluR2-lacking AMPARs. However, according to our data, the increase in current amplitude during PdF under NOS inhibition disappears, which indicates an unlikelihood of an increased concentration of intracellular polyamines in apical dendrites.

Nitric oxide may also act through the regulation of CP-AMPAR trafficking or through the modification of incorporated receptors. NO inhibition could disrupt one of the possible mechanisms involved in the trafficking of AMPAR subunits: the indirect sGC-dependent pathway (Serulle et al., 2007), direct nitrosylation of GluR1 subunits (Selvakumar et al., 2013; Von Ossowski et al., 2017), or different protein–protein interactions (Chen et al., 2000; Zhang et al., 2015; for review, see Ivanova et al., 2020). One such interaction was shown for GluR2 incorporation *via* NSF-dependent declustering of the PICK1–GluR2 complex (Hanley et al., 2002; Huang et al., 2005; Sossa et al., 2007); however, in the two latter studies the NO donor application resulted in increased GluR1 surface expression (Hanley et al., 2002, Figure 5B and Sossa et al., 2007, Figure 4C); thus, there is a possibility of NSF involvement in GluR2-lacking AMPAR trafficking. We tested the cGMP-dependent pathway by blocking sGC (Figure 6) but did not find any differences in the rectification characteristics of the studied synapses.

Obtained results suggest that nitric oxide upregulates the CP-AMPAR sensitivity to polyamines: this might explain changes in the current–voltage relationships, decreased PdF, and persisting EPSC growth under NOS inhibition. Moreover, such modulation was demonstrated for GluR2-lacking AMPARs by the auxiliary protein stargazin (Soto et al., 2007). However, the mechanism of this modulation requires further clarification. One possible explanation of the NOS inhibition effect is a reduced GluR2-lacking AMPAR surface expression. However, that does not explain the increase in EPSC amplitudes during polyamine washout after incubation in L-NAME.

In conclusion, this study demonstrates the effects of NOS inhibition on GluA2-lacking AMPA receptor-mediated currents at apical but not basal dendrites of the CA1 pyramidal neurons. This effect could underlie the differences in synaptic plasticity of the aforementioned synapses, although the mechanisms of this effect require further study. Many studies demonstrated differences in the mechanisms of synaptic plasticity between different neuron’s compartments; the concept of a specialized “memory synapse” is discussed (Sossin, 2018). We believe that our study highlights the importance of such phenomena as synaptic heterogeneity which may underlie the features of information processing in the hippocampus. In addition, the importance of AMPA receptors for such aspects of cell life as synaptic plasticity and homeostasis is undeniable. AMPAR GluA1–4 subunit trafficking, subunit-specific protein interactions, auxiliary subunits, and posttranslational modifications could predict the types and extent of synaptic plasticity; this is the so-called “AMPA receptor code of synaptic plasticity” (Diering and Huganir, 2018), and our data reveal more details of this complex code.

## Data Availability Statement

The raw data supporting the conclusions of this article will be made available by the authors, without undue reservation.

## Ethics Statement

The animal study was reviewed and approved by the Ethical committee of the Institute of Higher Nervous Activity and Neurophysiology, Russian Academy of Sciences (IHNA RAS).

## Author Contributions

VI designed and performed the experiments, analyzed the data, and wrote the article. PB managed the project and wrote the article. NB conceived and designed the experiments, managed the project, and wrote the article. All authors contributed to the article and approved the submitted version.

## Conflict of Interest

The authors declare that the research was conducted in the absence of any commercial or financial relationships that could be construed as a potential conflict of interest.
