# Supplementary material for: Nitric Oxide Regulates GluA2-Lacking AMPAR Contribution to Synaptic Transmission of CA1 Apical but Not Basal Dendrites
Source: Front Synaptic Neurosci. 2021 Jun 3;13:656377. doi: 10.3389/fnsyn.2021.656377 (PMC8210775; doi:10.3389/fnsyn.2021.656377)
Supplement: Supplementary file 1 [file Data_Sheet_1.zip › Supplementary figure legends.docx]

Supplementary figure 1. Comparison of the rectification indices (RIs) registered with 10µM and 100µM spermine in the intracellular solution. RIs of the apical and basal dendrites registered with 100µM spermine in the intracellular solution were not significantly different. Open gray circles represent individual data points.

Supplementary figure 2. Polyamine-dependent facilitation (PdF) registered ~1minute, 5 minutes and 20 minutes after whole-cell formation using spermine-free intracellular solution. Dendrites in str. oriens and in str. radiatum have different morphology and washout of polyamines via patch pipette from the dendrites in str. oriens occurs faster.

Supplementary figure 3. Comparison of basal transmission registered with spermine-free (purple and orange) and 10µM spermine-containing intracellular solution (black and blue). Presence of 10µM spermine in the patch pipette did not cause increase of the EPSC amplitude.
