# Supplementary figures and images for: Nitric Oxide Regulates GluA2-Lacking AMPAR Contribution to Synaptic Transmission of CA1 Apical but Not Basal Dendrites
Source: Front Synaptic Neurosci. 2021 Jun 3;13:656377. doi: 10.3389/fnsyn.2021.656377 (PMC8210775; doi:10.3389/fnsyn.2021.656377)

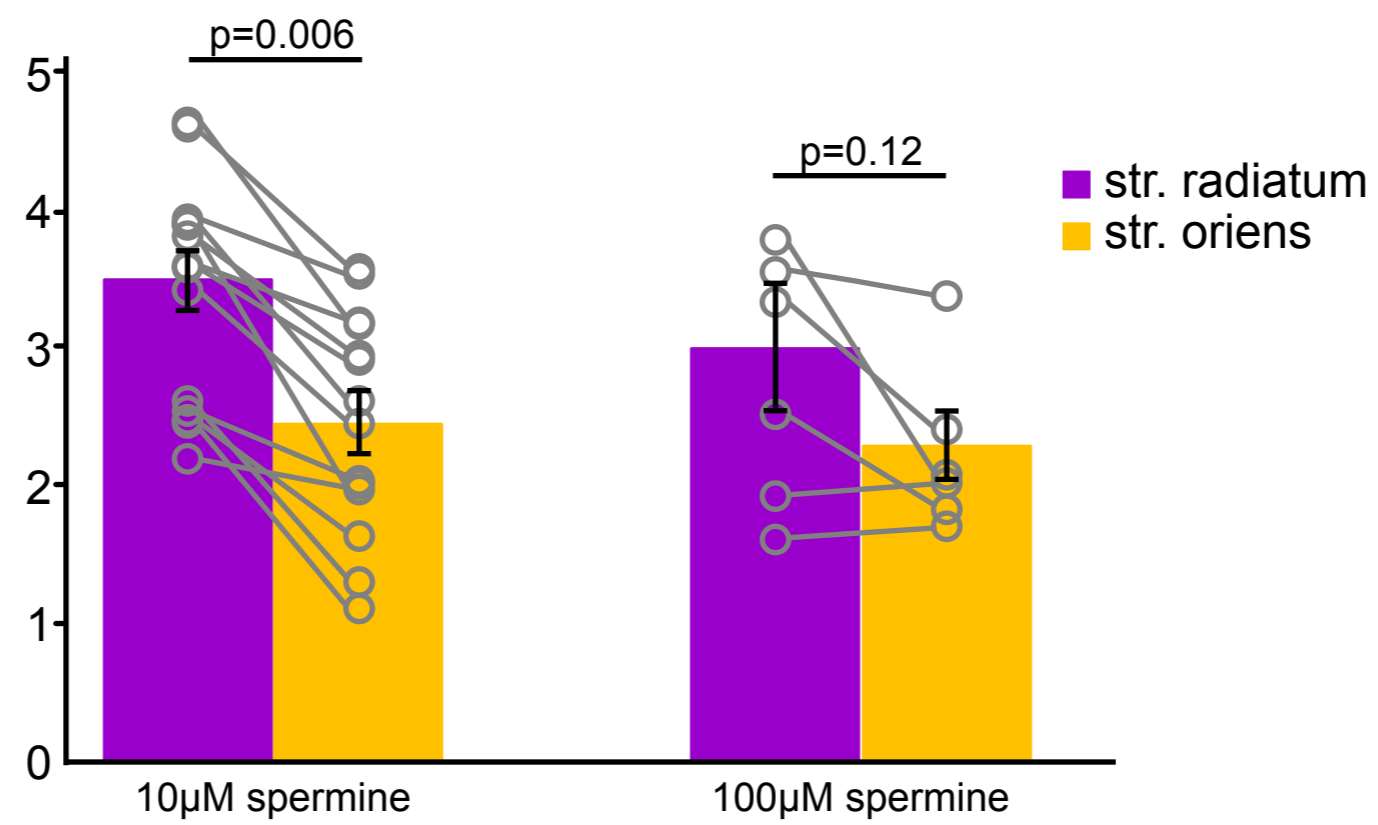

Supplementary Figure 1

Supplement: Supplementary file 1 [file Data_Sheet_1.zip › suppl 1.pdf]

## str. radiatum

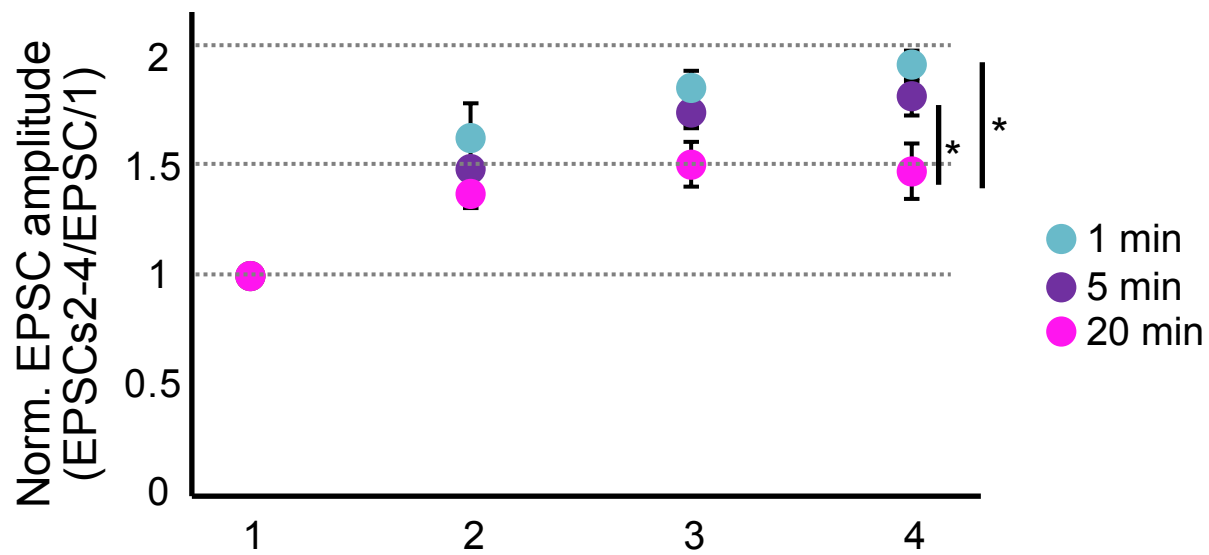

## str. oriens

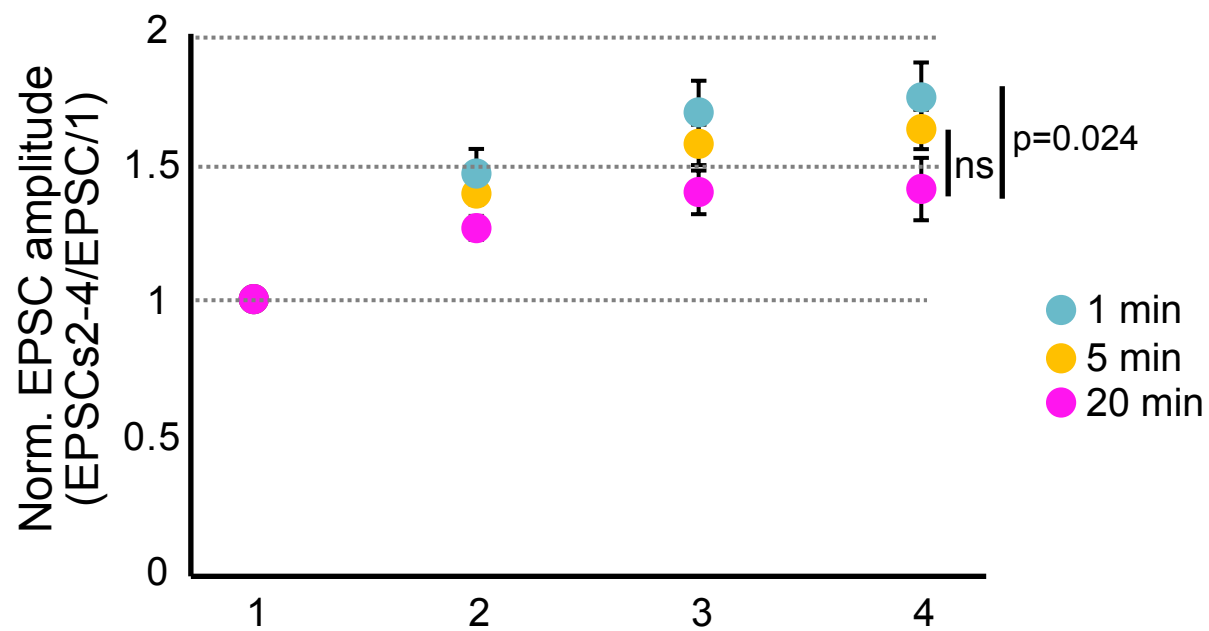

Supplementary Figure 2

Supplement: Supplementary file 1 [file Data_Sheet_1.zip › suppl 2.pdf]

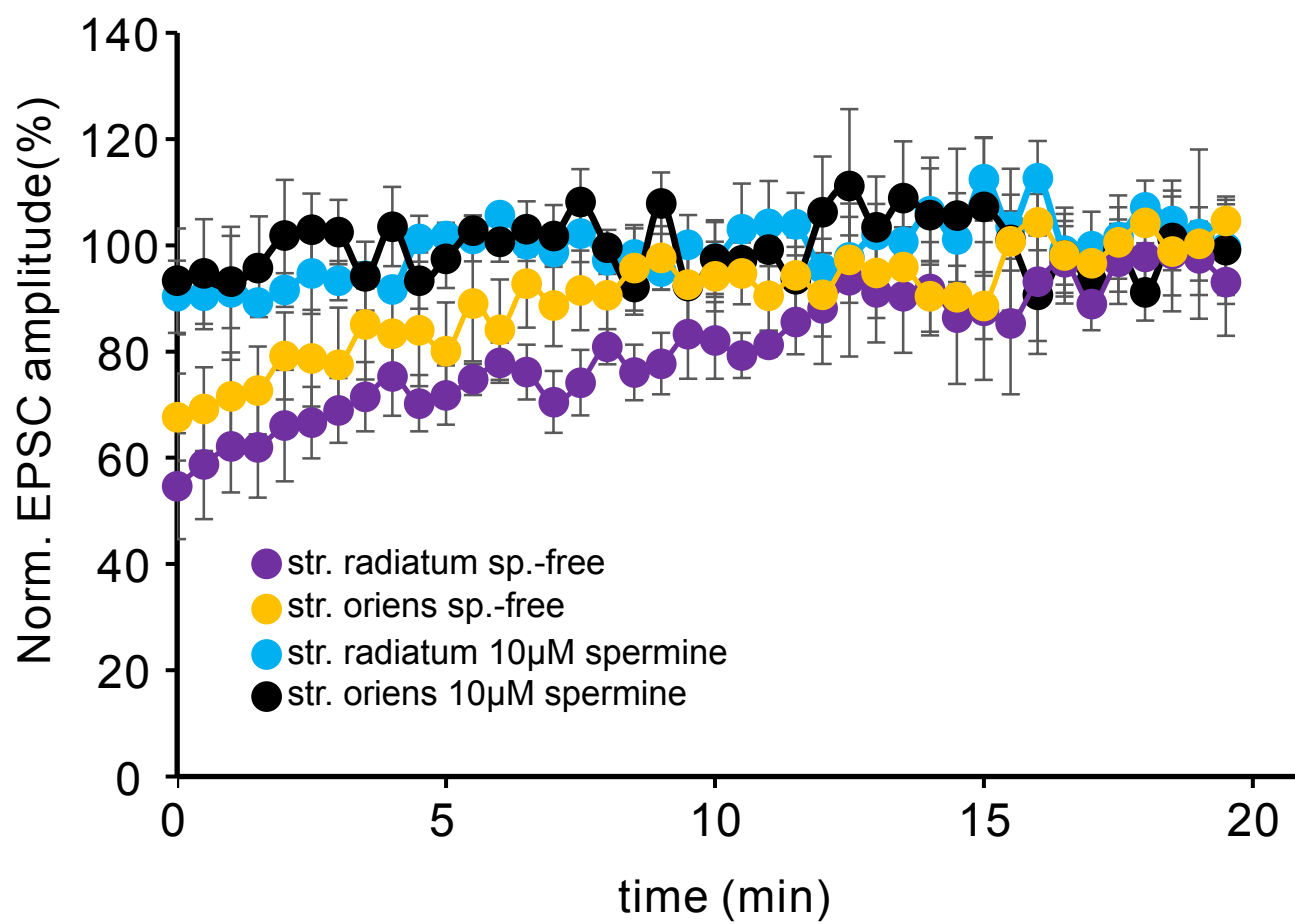

Supplementary Figure 3

Supplement: Supplementary file 1 [file Data_Sheet_1.zip › suppl 3.pdf]
